# Starch–Glycerol-Based Hydrogel Memristors for Bio-Inspired Auditory Neuron Applications

**DOI:** 10.3390/gels11060423

**Published:** 2025-06-01

**Authors:** Jiachu Xie, Yuehang Ju, Zhenwei Zhang, Dianzhong Wen, Lu Wang

**Affiliations:** School of Electronic Engineering, Heilongjiang University, Harbin 150080, China

**Keywords:** memristor, hydrogel, starch–glycerol, artificial auditory neuron, spatiotemporal integration, gain modulation, synchronization detection

## Abstract

In the era of artificial intelligence, the demand for rapid and efficient data processing is growing, and traditional computing architectures are increasingly struggling to meet these needs. Against this backdrop, memristor devices, capable of mimicking the computational functions of brain neural networks, have emerged as key components in neuromorphic systems. Despite this, memristors still face many challenges in biomimetic functionality and circuit integration. In this context, a starch–glycerol-based hydrogel memristor was developed using starch as the dielectric material. The starch–glycerol–water mixture employed in this study has been widely recognized in literature as a physically cross-linked hydrogel system with a three-dimensional network, and both high water content and mechanical flexibility. This memristor demonstrates a high current switching ratio and stable threshold voltage, showing great potential in mimicking the activity of biological neurons. The device possesses the functionality of auditory neurons, not only achieving artificial spiking neuron discharge but also accomplishing the spatiotemporal summation of input information. In addition, we demonstrate the application capabilities of this artificial auditory neuron in gain modulation and in the synchronization detection of sound signals, further highlighting its potential in neuromorphic engineering applications. These results suggest that starch-based hydrogel memristors offer a promising platform for the construction of bio-inspired auditory neuron circuits and flexible neuromorphic systems.

## 1. Introduction

Since Leon Chua proposed the concept of the memristor in 1971, it has evolved from a theoretical discussion to an electronic component of significant practical value [1,2,3]. In recent years, memristors have attracted widespread attention due to their unique nonvolatile memory characteristics and their ability to mimic biological synaptic functions [4,5,6]. Memristors not only provide new ideas for solving the bottleneck problems of von Neumann architecture but also pave new paths for the development of intelligent computing and sensing systems [7,8]. The resistance of a memristor changes with the amount of current passing through it, and the corresponding resistance can be maintained. This characteristic is very similar to the change in synaptic weight of biological synapses under biological–electrical signal stimulation, which has sparked great interest in research on synaptic biomimetic devices based on memristors [9,10,11,12].

Threshold-type memristor devices can rapidly switch from a high-resistance state to a low-resistance state after reaching a specific threshold voltage [13,14]. An ideal threshold-type memristor exhibits very typical gating characteristics and memristive properties. Threshold-switching memristors based on the conductive filament mechanism have a working mechanism similar to that of ion channels in neurons [15,16,17]. When building neuron circuits with volatile threshold-switching devices, they can spontaneously reset themselves like biological neurons, thus eliminating the need for auxiliary reset circuits that are relied upon in neuron modules based on nonvolatile memristors [18,19]. More importantly, the random threshold-switching behavior of conductive filament formation is particularly suitable for mimicking the stochastic firing characteristics of biological neurons [20].

In recent years, the development of threshold-type memristor technology has laid the foundation for building complex and efficient neuromorphic computing systems [21,22]. In parallel with hardware-based implementations, recent research has also highlighted the potential of wetware systems in mimicking neural functions. For example, nonlinear chemical systems, such as the Belousov–Zhabotinsky (BZ) reaction, can form spatiotemporal chemical wavefronts to emulate cochlear-like auditory information processing [23]. Similarly, kombucha–proteinoid hydrogel networks, developed by integrating microbial cultures with synthetic biological elements, have been shown to produce oscillatory electrical responses to audio stimuli, suggesting proto-neural computation capabilities [24]. These approaches offer biologically inspired and energetically efficient alternatives for neuromorphic signal transduction. By referencing both solid-state and biochemical systems, a more holistic understanding of artificial auditory function can be established. Wen and others constructed artificial visual neurons using resistors and threshold-switching memristors, achieving rate coding by modulating pulse parameters and the resistance of R_s_ [25]. Wang and colleagues fabricated a selector-one resistor unit composed of a selector and an RRAM based on egg albumen, effectively solving the leakage current problem in crossbar arrays [26]. Khan and his team prepared a nickel-doped zinc oxide/gold-based threshold memristor for artificial nociceptors that simulates nociceptive behaviors, such as pain thresholds, relaxation, allodynia, and hyperalgesia [27]. Sokolov developed a threshold-type memristor with an Ag/Ti_3_C_2_T_x_NS/Pt structure, which realized some synaptic behaviors of biological synapses [28].

The research and development of auditory neurons has always been an important direction in the fields of neuroscience and neural engineering. Auditory neurons are responsible for converting the sound waves they receive into electrical signals that the brain can understand and recognize sounds through complex spatiotemporal information integration [29,30,31]. Although traditional technologies have made some progress in simulating these processes, there are still issues such as excessive device size and low energy efficiency [32]. The development of memristor technology has made it possible to simulate more complex and efficient auditory neurons. Memristors can be used to construct more compact and energy-efficient artificial neurons and synapses, which not only helps in understanding the working mechanism of the auditory system but also provides an important technical foundation for designing new types of intelligent auditory systems [33,34,35].

Starch–glycerol–water mixtures have been widely reported to form physically cross-linked hydrogel systems, exhibiting characteristics such as three-dimensional polymer networks, high water content, and mechanical flexibility [36,37]. Qamruzzaman et al. systematically classified starch-based hydrogels as physically cross-linked networks with high water retention and environmental adaptability, supporting their recognition as soft matter platforms in bioelectronics [36]. Shang et al. further confirmed that starch hydrogels possess inherent three-dimensional network structures formed via dual physical crosslinking, validating their definition as stable hydrogel systems suitable for neuromorphic device construction [37]. In this research, we developed a threshold-type biological memristor using starch as the dielectric layer, which exhibits a large current switching ratio and stable threshold voltage. These characteristics make it highly promising for applications in simulating neuronal behavior. Furthermore, by using this type of threshold memristor in parallel with a capacitor and then in series with a load resistor, we successfully constructed an auditory neuron that simulates spiking behavior. The characteristics of the spiking neuron were modulated by adjusting the pulse and circuit parameters. Additionally, using this device, we achieved gain modulation and synchronous detection of different input sound signals, demonstrating its immense potential in neuromorphic engineering. It should be noted that the term “artificial auditory neuron” used in this work refers specifically to the emulation of auditory neuronal response behaviors via electrical pulse stimulation, and does not involve actual acoustic signal acquisition. This approach is consistent with established practices in neuromorphic device research.

## 2. Results and Discussion

**Figure 1:**
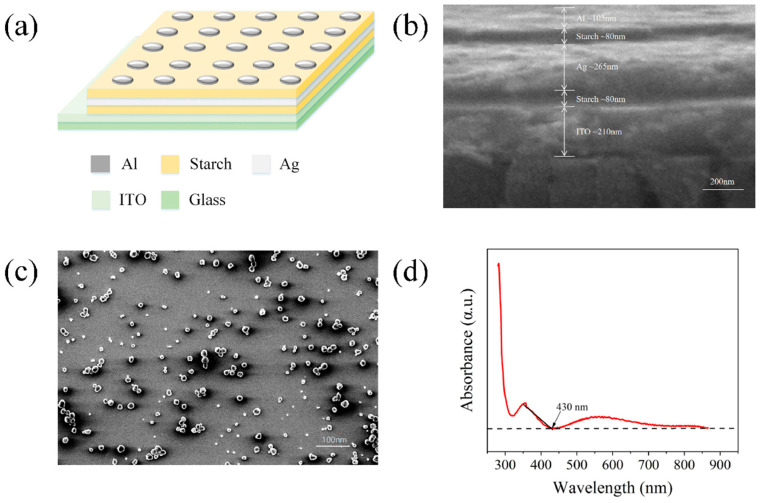
(**a**) Structural diagram of the Al/starch/Ag/starch/ITO memristor. (**b**) Cross-sectional SEM image of the device. From top to bottom: Al top electrode (~103 nm), upper starch layer (~80 nm), Ag layer (~265 nm), lower starch layer (~80 nm), ITO (~210 nm), and the glass substrate. (**c**) Top-view SEM image of the upper starch film, demonstrating a uniform and compact surface morphology. (**d**) UV–visible absorption spectrum of starch.

As shown in Figure 1a, the basic structure of the threshold-type memristor consists of a substrate made of 20 mm × 20 mm × 1.1 mm glass with an ITO conductive layer. The device is composed, from top to bottom, of an Al (aluminum) top electrode, a starch film, an Ag (silver) film, another starch film, and an ITO film. As shown in Figure 1b,c, the structure of the memristor device was examined using scanning electron microscopy (SEM). Figure 1b presents the cross-sectional image of the device, where the layers from top to bottom are as follows: Al top electrode (~103 nm), upper starch layer (~80 nm), Ag conductive layer (~265 nm), lower starch layer (~80 nm), ITO bottom electrode (~210 nm), and the glass substrate. The interfaces are clearly defined, and the layer thicknesses are uniform and well controlled, indicating a stable fabrication process and good structural integrity. Figure 1c shows the top-view SEM image of the surface morphology of the upper starch layer. The film surface is continuous and well-defined, with no signs of aggregation or cracking. A number of discrete bright spots are observed across the surface, which may result from localized microstructure formation or partial recoagulation during the drying process. These features are characteristic of the nanoscale heterogeneity commonly observed in physically cross-linked hydrogel films, and do not indicate defects. The overall morphology remains consistent and stable, which supports the device’s good electrical performance and repeatability. Moreover, such surface characteristics align well with previous reports on starch-based hydrogel films, which typically form dense polymer networks with uniform hydration patterns [37].

The UV–visible absorption spectrum of the starch thin film is shown in Figure 1d. The measurement was performed on a single starch dielectric layer, representative of both the upper and lower starch films in the device due to identical fabrication parameters. The absorption edge was identified at a wavelength (λ) of 430 nm. Based on the equation Eg = hc/λ, the optical band gap of the starch material was calculated to be 2.88 eV. The sharp absorption edge and nearly linear increase in absorbance with photon energy suggest that the starch layer exhibits a direct-type optical band gap, consistent with the direct-transition model proposed by classical optical absorption theory [38]. This band gap is attributed to the starch dielectric layer, which dominates the absorption behavior in the visible region.

**Figure 2:**
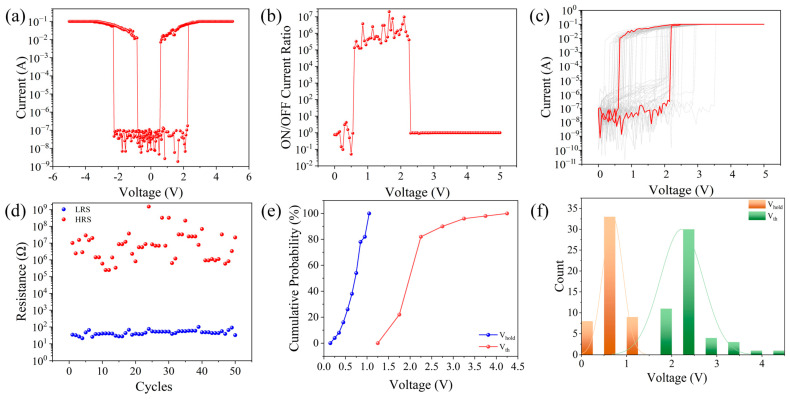
(**a**) I–V characteristic curve of the Al/starch/Ag/starch/ITO device, (**b**) switching current ratio, (**c**) I–V characteristic curve of 50 cycles, (**d**) device durability, (**e**) cumulative probability of threshold voltage distribution, and (**f**) threshold voltage distribution of the device.

The I–V characteristics of the memristor are shown in Figure 2a, displaying threshold-type resistive switching properties. During the testing process of the memristor, the bottom electrode ITO was grounded, and a bias voltage was applied to the top electrode Al. The chosen scanning method for this device’s switching characteristics was a bias voltage of 0 V → 5 V → −5 V → 0 V, resulting in the I–V characteristic curve shown in Figure 2a. When a bias of 0 V → 5 V was applied, the initial state of the device was a high-resistance state (HRS). At an applied bias voltage of 2.3 V, the current instantaneously increased from 1.77 × 10^−7^ A to 0.07 A, transitioning the device from high to low resistance. Subsequently, when a bias of 0 V → 5 V was applied at a voltage of 0.55 V, the current abruptly changed from 7.12 × 10^−3^ A to 5.21 × 10^−9^ A, shifting the device from the LRS to the HRS. Similarly, when a bidirectional scanning voltage of 0 V → −5 V → 0 V was applied, the device also completed the HRS → LRS → HRS transition. Figure 2b shows the device’s switching current ratio, with the Al/starch/Ag/starch/glass memristor having a maximum switching current ratio of 2 × 10^7^. To study the device’s repeatability, a unit from the top electrode array of the Al/starch/Ag/starch/ITO/glass device was selected, and 50 consecutive DC voltage sweeps were applied. The I–V curves in Figure 2c demonstrate that the memristor exhibits generally stable switching behavior under ambient conditions, although some degree of fluctuation is observed, which can be attributed to the intrinsic variability of bio-based materials. While not as uniform as inorganic systems, the starch-based device maintains clear threshold-switching and reset characteristics, supporting its feasibility for neuromorphic applications. Figure 2d shows how the resistance values of the HRS and LRS change with the number of cycles under a voltage of 1 V. The LRS resistance of the device is relatively stable. To analyze the threshold voltage distribution of the memristor, a threshold voltage distribution diagram was drawn, as shown in Figure 2e, as well as a cumulative probability chart of the threshold voltage distribution at 1 V, as shown in Figure 2f. The threshold voltage distribution of the device is relatively concentrated.

**Figure 3:**
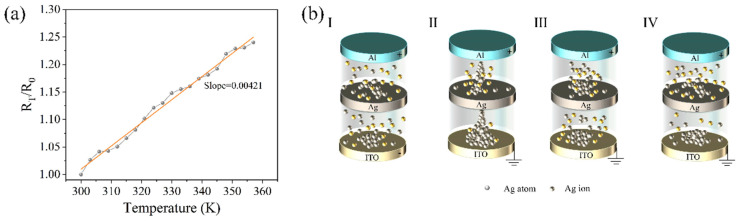
Conductive mechanism of the device. (**a**) Relationships between resistance and temperature. (**b**) Conductive mechanism model of the device.

Further tests were conducted to examine the change in the resistance of the device with temperature, as shown in Figure 3a. Here, R_0_ represents the resistance at a temperature of 300 K, and R_T_ represents the resistance at temperature T. The slope in the graph is approximately 4.21 × 10^−3^, suggesting that the resistance switching is caused by the formation and rupture of Ag conductive filaments in the dielectric layer. The conductive mechanism model of the device is depicted in Figure 3b. When no bias is applied, the particles in the dielectric layer are randomly distributed, and the device is in a high-resistance state (HRS), as shown in Figure 3b-I. When a positive bias is applied to the top electrode, the Ag atoms in the middle silver layer are oxidized into Ag ions under the influence of the electric field. These ions migrate toward the bottom electrode in the dielectric layer and capture electrons to reduce back to atoms, leading to the accumulation of atoms. As the external voltage increases, the electric field intensifies, and the local Joule heat under high current enhances the migration rate of cations. When enough silver atoms accumulate, they form conductive filaments connecting the top electrode to the bottom electrode in the dielectric layer, significantly increasing the device’s conductivity and shifting it to the low resistance state (LRS), as shown in Figure 3b-II. However, the current passing through these small local conductive filaments generates a large amount of Joule heat, causing a rapid increase in the temperature of the filaments and leading to their rupture. At this point, the resistance between the Ag layer and the ITO electrode increases, as shown in Figure 3b-III. Additionally, as the external bias voltage decreases, due to Coulomb repulsion, Ag ions are pushed back, and the entire device switches back to the HRS, as depicted in Figure 3b-IV.

**Figure 4:**
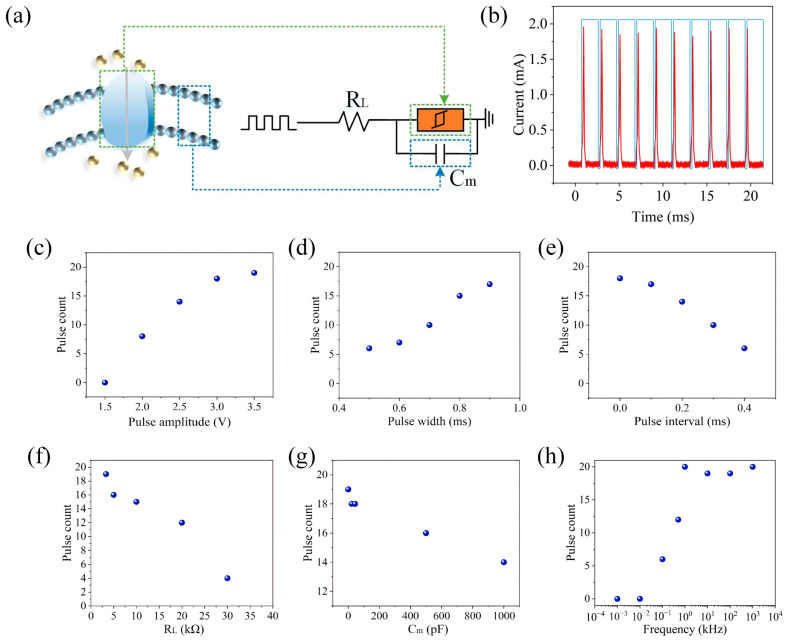
Memristor-based artificial neuron construction. (**a**) Schematic diagram of artificial neurons. (**b**) Spike discharge behavior of artificial neurons. The pulse and circuit parameters were adjusted to adjust the characteristics of spiking neurons: (**c**) change in pulse amplitude, (**d**) change in pulse width, (**e**) change in pulse interval, (**f**) change in resistance, (**g**) change in capacitance, and (**h**) change in input frequency.

The spiking neuron can be implemented through the circuit shown in Figure 4a, where the threshold-type memristor is connected in parallel with a capacitor (C_m_) and then connected in series with a load resistor (R_L_). The threshold-switching behavior of the device can mimic the dynamic switching of ion channels near the soma of a neuron. Figure 4b displays the peak behavior of the artificial neuron, where the pulse sequence has a pulse width of 1.9 ms and an amplitude of 3 V, with an interval of 0.1 ms between adjacent pulses. Due to the voltage division effect, Cm initially charges up because the voltage primarily drops across the device (R_HRS_ > R_L_). Once the voltage across the capacitor reaches Vth, the device switches from the HRS to the LRS, representing the discharge of the artificial spiking neuron, which is observed as a current peak. After the device switches to the ON state, the voltage between the R_L_ and the device redistributes, and the capacitor begins to discharge. When the voltage drops below Vhold, the device returns to the HRS state. As shown in Figure 4c–h, the characteristics of the spiking neuron can be modulated by adjusting the pulse and circuit parameters. In biological neurons, the peak frequency increases with increasing input stimuli. To simulate this behavior, a series of pulse sequences with different pulse amplitudes of 1.5, 2, 2.5, 3, and 3.5 V and a pulse width of 1.9 ms (with an interval of 0.1 ms), as well as pulse sequences at the same frequency but with pulse widths of 0.5, 0.6, 0.7, 0.8, and 0.9 ms (and an amplitude of 3 V), were applied to the artificial neuron. The results indicate that the pulse frequency increases with increasing pulse amplitude or pulse width due to the increased overall charging speed, demonstrating that the firing frequency of the artificial auditory neuron can be flexibly regulated through electrical parameters, similar to the frequency modulation seen in biological neurons. Additionally, due to the charge leaking through the device parallel to the C_m_, the peak frequency decreases with increasing pulse intervals, thus realizing the leaky dynamics of the neuron, as shown in Figure 4c–e. Furthermore, the relationship between the number of pulses and the circuit parameters was tested. The results showed that pulse counting decreases with increasing R_L_ and C_m_, as illustrated in Figure 4f–h. Additionally, pulse counting increases with increasing input frequency and reaches saturation at very high frequencies, as shown in Figure 4h.

**Figure 5:**
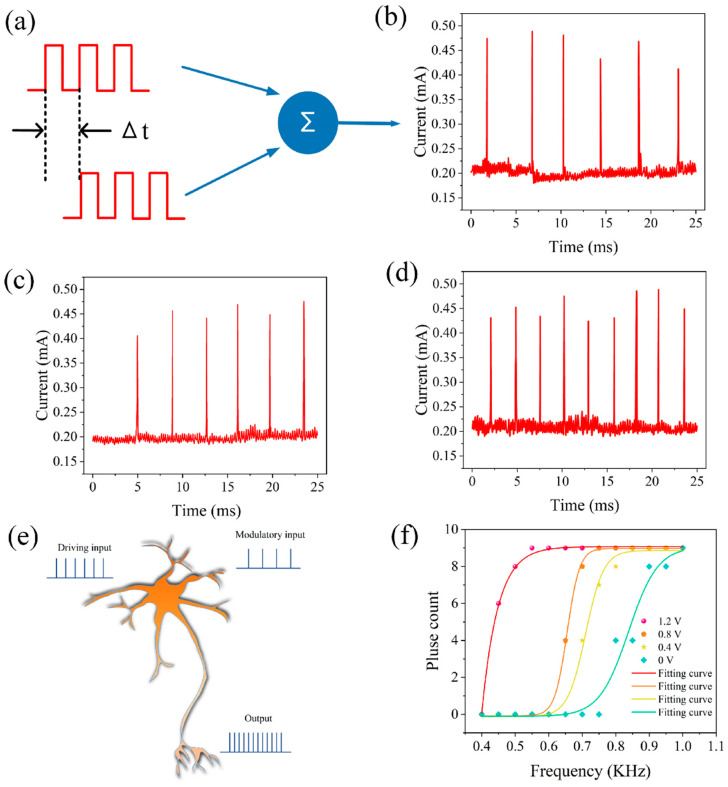
Spatiotemporal integration of two pulse sequences at different time differences. (**a**) Schematic diagram of spatiotemporal integration. (**b**) Δt = 2.2 ms (**c**) Δt = −2.2 ms. (**d**) Δt = 0. Artificial auditory neurons realize neuron gain modulation. (**e**) Schematic diagram of neuron gain modulation. (**f**) The function of the number of neuron pulses with respect to the driving input frequency.

Neurons can also perform spatiotemporal summation of input information, as illustrated in Figure 5a. When two temporally correlated pulse sequences were applied (amplitude 1 V, width 1.9 ms, interval 0.1 ms, repeated 10 cycles), the discharge frequency of the artificial neuron was also a function of the time interval between the two pulse sequences. When the time difference was 2.2 ms (*Δt* = 2.2 ms), the neuron had six spike discharges within the same time period (25 ms), as shown in Figure 5b. Similarly, when the time difference was −2.2 ms (*Δt* = −2.2 ms), the neuron also had six spike discharges, as shown in Figure 5c. However, when the pulses were applied simultaneously, the neuron had nine spike discharges, as illustrated in Figure 5d.

Traditionally, the brain’s powerful computing ability has been attributed to its complex neural network connections, where neurons perform complex nonlinear operations, converting synaptic inputs into output signals. These nonlinear mechanisms enable neurons to carry out a range of computations, and the realization of artificial neurons with this capability is significant for building neuromorphic systems. Here, we demonstrate that neurons can achieve neural modulation, as illustrated in Figure 5e. The output of a neuron is influenced by two types of presynaptic inputs: one is the driving input, which enables the associated neurons to discharge strongly; the other is the modulatory input, which regulates the effectiveness of the driving input. By observing the output of a neuron under the combined action of modulatory and driving inputs, we can determine whether the neuron has the capability of gain modulation. This kind of gain modulation is often observed in the responses of cortical neurons and is believed to play an important role in neural computation. Figure 5f shows the function of the number of spikes of a neuron against the frequency of the driving input at different amplitudes, indicating the realization of neural gain modulation.

**Figure 6:**
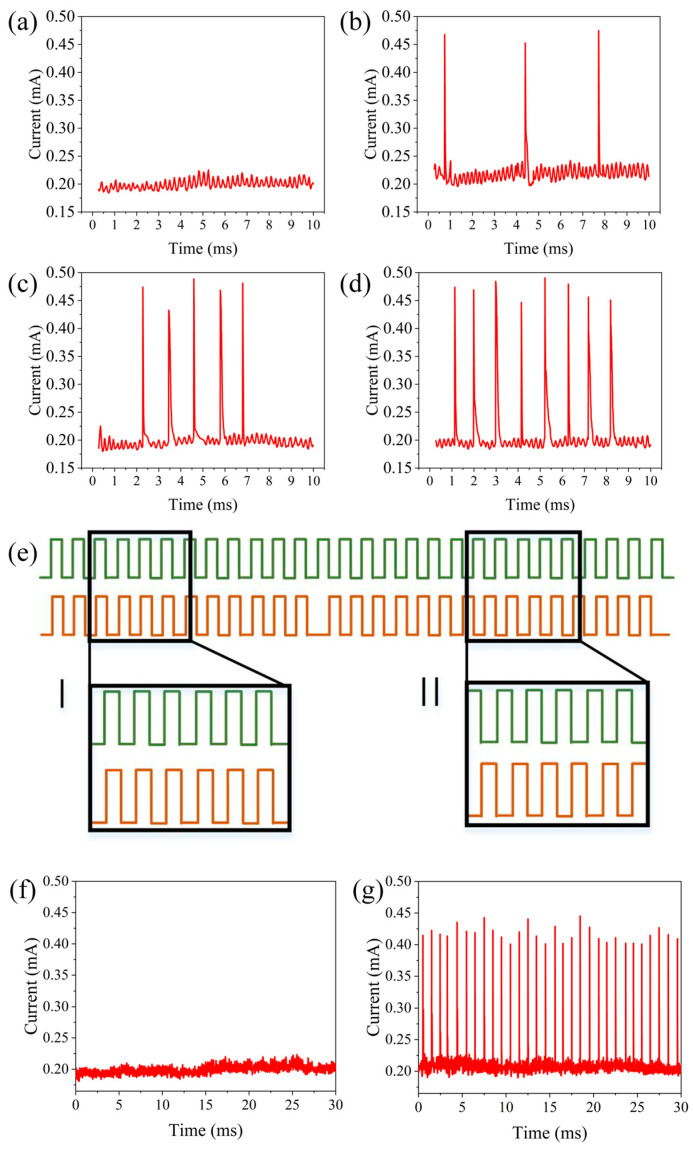
Neuron output with different modulation input amplitudes: (**a**) amplitude of 0 V, (**b**) amplitude of 0.4 V, (**c**) amplitude of 0.8 V, (**d**) amplitude of 1.2 V. Synchronous detection based on artificial auditory neurons: (**e**) schematic diagram of the input pulse, (**f**) neuron output when the input is synchronized, and (**g**) neuron output when the input is not synchronized.

To achieve similar functionality, a series of pulses (amplitude 1 V, width 0.5 ms, interval 0.5 ms, and repeated for 10 cycles) were applied as the driving input to the aforementioned neuron, and another pulse sequence (amplitudes of 0, 0.4, 0.8, and 1.2 V, width of 0.5 ms, interval of 0.5 ms, and repeated 10 times) served as the modulatory input. The discharge spike count was defined as the neuron’s output. Figure 6a–d show the neuron’s output for modulatory inputs at amplitudes of 0, 0.4, 0.8, and 1.2 V combined with the driving input. With the driving input alone, the neuron’s discharge spike count was 0, but with additional modulatory inputs at 0.4 V, 0.8 V, and 1.2 V amplitudes, the spike counts of the neuron were three, five, and eight, respectively. Synchronous detection has been found to be an efficient information processing function that is significant in the auditory and visual systems. This type of synchronous detection can be achieved based on the spatiotemporal dynamics of neurons, as shown in Figure 6e. As illustrated in Figure 6f, when two presynaptic inputs (amplitude 1 V, width 0.5 s, interval 0.5 s, continued for 30 cycles) are applied simultaneously, the output neuron is activated at a discharge rate of ~1 kHz. When the two presynaptic inputs are asynchronous, the neuron fails to discharge, as shown in Figure 6g. These test results are consistent with the spatiotemporal dynamic characteristics of neurons, indicating the potential of this neuron for synchronous detection. Based on these characteristics, it can be utilized to detect moving sound sources from the front and rear directions.

## 3. Conclusions

This study tested and analyzed the electrical performance of a threshold-type biological memristor with starch as the dielectric layer. The results show that the device has a large current-to-switch ratio and a stable threshold voltage. This performance is mainly attributed to the formation and breakage of Ag conductive filaments inside the device. Moreover, the device demonstrated great potential in simulating neuronal behavior. Using this memristor, an auditory neuron was constructed, and the experimental results indicate that this neuron can achieve spike discharge and leaky dynamics. Additionally, based on this artificial auditory neuron, functions such as spatiotemporal summation of input information, gain modulation, and synchronous detection of sound signals were successfully implemented.

In addition to demonstrating basic neuronal behaviors at the device level, future efforts will focus on exploring the scalability of the starch-based memristor platform. The fabrication process is solution-based and conducted at low temperatures, making it suitable for large-area processing, as well as compatible with flexible substrates. Moreover, the starch–glycerol–water formulation used here has been classified in literature as a physically cross-linked hydrogel, characterized by mechanical flexibility and a three-dimensional polymer network [36]. These hydrogel properties further support the formulation’s compatibility with soft electronics and bio-integrated systems. The biocompatibility and biodegradability of starch offer further advantages for short-term, eco-friendly, and possibly implantable neuromorphic systems. These features suggest the potential for integration into larger neuromorphic circuits and networks. Future work will explore array-level device configuration, crossbar interconnection, and hybrid architectures with inorganic elements to facilitate system-level applications.

Despite these promising outcomes, several key challenges remain for transitioning starch-based threshold-type memristors toward practical neuromorphic systems. First, device-to-device variability persists due to the intrinsic heterogeneity of biopolymer films. Second, the environmental sensitivity of starch, especially under humidity and temperature fluctuations, may impact switching consistency. Third, although the device demonstrates good volatile switching behavior, its cycle endurance and operational stability over time still require further optimization. Addressing these limitations will involve efforts in material optimization, encapsulation strategies, and hybrid integration with stable inorganic interfaces. These future directions will be crucial for improving device reliability, reproducibility, and system-level feasibility in bio-memristive neuromorphic platforms.

## 4. Materials and Methods

To prepare the threshold-type biological memristor with the structure of Al/starch/Ag/starch/indium tin oxide (ITO)/ glass, the following steps were followed: The glass substrate coated with ITO electrodes was ultrasonically cleaned in acetone, ethanol, and deionized water for 15 min, after which 0.4 g of starch was weighed and mixed with 2 mL of deionized water and 8 mL of glycerol. This mixture was magnetically stirred for 5 h to obtain a homogeneous starch–glycerol–water solution, previously recognized in literature as a physically cross-linked hydrogel system [36,37]. The starch solution was then spin-coated onto the ITO/glass substrate, initially at a speed of 500 rpm for 5 s, followed by heating at 2000 rpm for 20 s. This formed a starch film, which was then dried at 80 °C for 10 min. Ag (silver) was deposited as an intermediate layer using the vacuum evaporation method under a vacuum of 2 × 10^−3^ Pa. The starch solution was then spin-coated again under the same conditions. Finally, Al (aluminum) electrodes were deposited over the starch layer using the vacuum evaporation method with a shadow mask, again under a vacuum of 2 × 10^−3^ Pa, followed by annealing at 80 °C for 10 min. This procedure completed the fabrication of the Al/starch/Ag/starch/ITO/glass threshold-type biological memristor.

## Data Availability

The datasets used and/or analyzed during the current study are available from the corresponding author upon reasonable request.
